# EDAER: Entropy-Driven Approach for Entity and Relation Extraction in Chinese Cyber Threat Intelligence

**DOI:** 10.3390/e28030261

**Published:** 2026-02-27

**Authors:** Yong Li, Xiuping Li, Yangbai Zhang, Zhiqiang Liu, Xiaowei Li, Qi Xu, Xiaolin Chang

**Affiliations:** 1 School of Cybersecurity, Northwestern Polytechnical University, Xi’an 100044, China; liyong1895@mail.nwpu.edu.cn (Y.L.); fly.669@163.com (X.L.); zhangyangbai1226@163.com (Y.Z.); 2 Data Communication Technology Research Institute, Beijing 100089, China; lixiaowei_bjut@163.com; 3 School of Cyberspace Science and Technology, Beijing Jiaotong University, Beijing 100006, China; 24120443@bjtu.edu.cn

**Keywords:** cross-entropy, entropy regularization, cyber threat intelligence, named entity recognition, entity relation extraction

## Abstract

Cyber threat intelligence (CTI) has been explored to strengthen system security via taking raw threat data from various data sources and transforming it into actionable insights that enable organizations to predict, detect, and respond to cyber threats. Named entity recognition (NER) and relation extraction (RE) are the key tasks of CTI data mining. However, current CTI NER and/or RE research is mainly focused on English CTI, which is not directly transferable to Chinese CTI due to fundamental linguistic and terminological differences. Moreover, the existing limited studies on Chinese CTI do not effectively address uncertainty in predictions in low-resource scenarios where entities and relations are sparse. This work aims to improve the performance of NER and RE tasks in low-resource Chinese CTI scenarios, and we make two major contributions. The first is that we construct a Chinese CTI dataset, which includes 16 types of entities and 9 types of relations—more than those of the existing open-source dataset on Chinese CTI. The second is that we propose an entropy-driven approach for entity and relation (EDAER) extraction. EDAER is the first to combine the techniques of RoBERTa_wwm, Mamba, RDCNN and CRF to perform NER tasks. In addition, EDAER is the first to apply entropy to quantify the uncertainty of the model’s predictions in NER and RE tasks in Chinese CTI scenarios. Moreover, EDAER is the first to apply contrastive learning techniques in Chinese CTI scenarios to learn meaningful features by maximizing the similarity between positive samples and minimizing the similarity between negative samples. Extensive experimental results on public and our built datasets demonstrate that our proposed approach performs the best. These results show that (1) RoBERTa_wwwm significantly outperforms BERT on both NER and RE tasks; (2) Mamba outperforms BiLSTM on the NER task; (3) the entropy-based dynamic gating mechanism contributes to performance improvements in both NER and RE tasks; and (4) the uncertainty-guided contrastive learning mechanism is helpful for performance improvement in the NER task.

## 1. Introduction

The global cybersecurity landscape deteriorated further in 2025, driven largely by a surge in zero-day exploits within advanced persistent threat (APT) campaigns [1,2]. Such threats present grave risks to national security, corporate intellectual property, and personal privacy. Cyber threat intelligence (CTI) has emerged as a critical line of defense [3]. By extracting actionable insights from heterogeneous unstructured sources, such as technical reports and news articles, CTI enables security analysts to anticipate adversary behavior and design proactive countermeasures.

**Figure 1:**
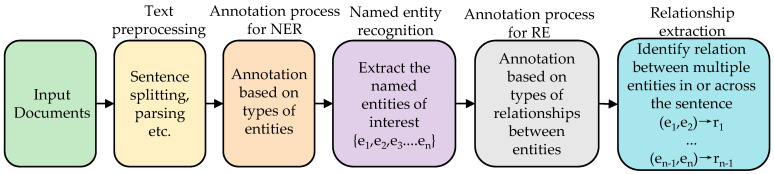
Pipeline showing associations between NER and RE.

A central mechanism for operationalizing CTI is the construction of cyber knowledge graphs (CKGs) [3]. CKGs integrate disparate threat data into a structured, queryable representation, thereby enhancing situational awareness and supporting the inference of potential attack trajectories. The construction of CKGs from unstructured text hinges on two fundamental natural language processing tasks: (1) named entity recognition (NER), which extracts and categorizes threat-relevant entities (e.g., vulnerability, software, organization), and (2) relation extraction (RE), which identifies semantic links between these entities. The past few years have witnessed substantial progress made in applying machine learning (ML) to these tasks, either jointly or in pipeline architectures [4]. Figure 1 illustrates the pipeline architecture used in our approach.

This work aims to improve the performance of NER and RE tasks in low-resource Chinese CTI scenarios. Here, “low-resource” refers to a scenario where there is a limited amount of high-quality, labeled data available to train ML models. Limited annotated data increases epistemic uncertainty, as the model cannot discern robust patterns from sparse examples, leading to poor predictive confidence.

There exist ML models for CTI-related NER and RE tasks, but most of them are designed for English CTI datasets [5,6]. Models for English CTI are not directly transferable to Chinese CTI due to fundamental linguistic and terminological differences. They exhibit much lower recognition accuracy for Chinese threat entities (e.g., “ransomware family names”, “IP addresses”) compared to English scenarios, failing to meet the needs of domestic cybersecurity defense. The authors in [5,6] considered Chinese CTI scenarios, but they did not take into account the low-resource characteristic. Furthermore, their work suffered from a limited scale and lack of diversity in labeled datasets.

This paper aims to address these issues. The two key contributions of this paper are summarized as follows.

(1) We propose an entropy-driven approach for entity and relation extraction (EDAER) tailored to low-resource Chinese CTI scenarios. EDAER consists of two components: the entropy-driven RoBERTa_wwm-Mamba-RDCNN-CRF (EDRMRC) model for identifying threat entities and the entropy-driven RoBERTa_wwm-RDCNN (EDRRC) model for extracting logical relationships between entity pairs generated by EDRMRC. Both models leverage entropy to quantify predictive uncertainty. Additionally, EDRMRC incorporates contrastive learning to improve the learning efficiency and enhance model robustness under low-resource conditions.

(2) We construct a Chinese CTI dataset, MyCDTier, covering 16 entity types and 9 relational categories, which addresses the insufficiency of existing datasets in entity coverage and relationship diversity.

The work in [6] is similar to ours but there are two major differences. The first difference is the dataset. We establish a dataset with greater entity coverage and relationship diversity. The second difference is that we apply new ML techniques, and we explore a solution for low-resource scenarios where entities and relations are sparse. We summarize the unique features of our proposed EDAER as follows.

(1) We are the first to combine the RoBERTa_wwm [7], Mamba [8], RDCNN [6], and CRF [6] machine learning techniques for the NER task. The combined model is denoted as RMRC in Section 5. The experimental results in Section 5 demonstrate the strength of this combination in named entity extraction. Note that EDRMRC consists of RMRC, entropy techniques, and contrastive learning techniques.

(2) We are the first to apply entropy in NER and RE tasks within Chinese CTI scenarios to quantify the uncertainty of model predictions. Additionally, we equip the model with an adaptive threshold to filter out low-confidence samples, thereby optimizing the training process and improving both the robustness and accuracy.

(3) We are the first to apply contrastive learning technology [9,10] in Chinese CTI scenarios to learn meaningful features by maximizing the similarity between positive samples and minimizing the similarity between negative samples. This helps the EDRMRC model to learn discriminative features that distinguish between different samples, enhancing the EDRMRC model’s robustness, particularly in low-resource environments where labeled data is limited.

We assess EDAER under both the public CDTier dataset [6] and our self-built MyCDTier dataset. All the experimental results validate the capabilities of EDAER.

The remainder of this paper is organized as follows. The existing related work is first discussed in Section 2 and then Section 3 details the problem. The proposed method is detailed in Section 4. Section 5 presents our experimental results and analyses. Finally, we summarize our work and give an outlook in Section 6.

## 2. Related Work

Significant studies on NER and RE tasks have been carried out. This section focuses on those for Chinese CTI. We first present the fundamentals of pretrained language models (PLM) in Section 2.1. Then, we present the existing works on NER and RE in Chinese CTI in Section 2.2 and Section 2.3. In each of the last two sections, we also present the techniques that have been applied to non-CTI datasets and will be applied in our approached proposed in this paper.

Table 1 summarizes the existing representative PLM-based NER and/or RE models for CTI, which are detailed in Section 2.2 and Section 2.3.

### 2.1. PLMs

Pretrained language models (PLMs) serve as powerful foundational models that significantly enhance performance across a wide range of natural language processing (NLP) tasks. This capability is achieved through unsupervised training on large-scale textual corpora. Both NER and RE are NLP tasks.

BERT [19], a foundational pretrained language model (PLM) introduced by Google in 2018, has been widely adopted to tackle numerous challenges in NLP. RoBERTa_wwm [7], an enhanced pretrained language model based on BERT, was proposed by Facebook AI Research (FAIR) to further advance NLP performance. In comparison to BERT, RoBERTa_wwm incorporates several key optimizations during pretraining, and it achieves superior performance across a wide range of NLP tasks.

### 2.2. Chinese CTI NER

Early research on Chinese CTI NER mostly focused on coarse-grained information extraction, relying primarily on keyword matching or text classification techniques. The CTI ANT system proposed in [20] identifies key terms related to attack techniques from Chinese APT reports and classifies threat intelligence types based on keyword matching with the MITRE ATT&CK framework. However, this system can only generate coarse-grained results such as “attack technique categories” and “keyword lists”. It fails to provide structured entity information like “attack organization names” and “vulnerability IDs”. A similar work [21] also has the same limitation—namely, its technical route leans more toward text classification and keyword extraction, without addressing the core needs of entity boundary detection and type judgment. Their output lacks structural characteristics, making it unsuitable for CKG construction.

Recent efforts have been devoted to fine-grained NER tasks, but they still mainly rely on generative or rule-aided approaches. Feng [22] proposed the PROMPT-BART model, which takes BART as the basic architecture and injects CTI domain knowledge through three complementary prompt designs, i.e., task prompts, example prompts, and template prompts, thus avoiding complex feature engineering. Although this approach simplifies the domain adaptation process, the generative model has the problem of “ambiguous entity boundaries” (e.g., incorrectly splitting “LockBit ransomware” into two entities: “LockBit” and “ransomware”). In addition, it relies on a large-scale prompt example library, resulting in insufficient generalizability in scenarios involving low-frequency threat entities.

With the breakthroughs of PLMs in semantic understanding, research on Chinese CTI NER has gradually shifted to the technical paradigm of PLM + domain-specific customization modules. The authors in [5] proposed the RoBERTa_wwm-RDCNN-CRF model, which employs RoBERTa_wwm to capture fine-grained semantics in Chinese CTI texts (such as industry-specific terms like “挂马 (malicious code embedding)” and “挖矿 (cryptocurrency mining)”). It integrates long-distance contextual information (e.g., cross-sentence associations between “attack organizations” and “vulnerabilities”) via residual dilated convolution (RDCNN) and finally optimizes the coherence of entity label sequences through a CRF layer. However, the parameter settings and ablation experiments for the RDCNN module have not been disclosed in detail. Additionally, it fails to adapt to the characteristic of “significant differences in entity spans” in Chinese CTI (for instance, “APT41” is a short entity, while “CVE-2024-XXXX remote code execution vulnerability” is a long entity), leaving room for improvement in the recognition accuracy for long entities. The authors in [6] constructed a Chinese CTI dataset, CDTier, and also investigated the capabilities of BERT-BiLSTM-GRU-CRF in NER tasks.

There have been studies on handling Chinese-English mixed entities. The authors in [12] designed a multi-task adversarial training model that takes “Chinese–English entity distinction” as an auxiliary task. Through an adversarial discriminator, the model’s semantic understanding of English terms such as “malware” and “zero-day” is enhanced. This approach effectively alleviates the problem of “English terms being misjudged as non-entities”, but the multi-task branch increases the model complexity. Its inference speed is 30% lower than that of single-task models, making it unable to meet the needs of real-time threat intelligence analysis.

All existing PLM-based NER models for Chinese CTI do not consider the low-resource characteristic. Furthermore, these works suffer from a limited scale and lack of diversity in labeled datasets. There have also been studies on class imbalance and low-resource adaptation. Liu et.al. [11] applied a Bayesian neural network to estimate the uncertainty of the predictions on a non-CTI dataset. The authors in [9,10] leveraged a focal loss function to address the class imbalance in NER tasks, but not for Chinese CTI. Recently, the technologies of uncertainty modeling and contrastive learning have been proposed for low-resource scenarios where there is a limited amount of high-quality, labeled data available to train ML models. See [13,14] and references therein. The core idea of contrastive learning is to train a model by pulling similar instances closer together and pushing dissimilar instances apart in the representation space. However, our investigation of the public literature indicated that no works have applied uncertainty modeling and contrastive learning for NER tasks on CTI datasets.

### 2.3. Chinese CTI RE

Early CTI RE research relied on manually constructed rules and keyword dictionaries. Although this enabled low-cost implementation, the generalizability was severely limited. For example, the keyword matching method based on the MITRE ATT&CK framework identifies relationships by presetting mapping rules such as “ exploit → attack organization—vulnerability” and “belong to → malware—family”. To address the scarcity of CTI annotated data, researchers have applied supervised learning and combined uncertainty modeling to improve the data utilization efficiency, among which entropy has been widely applied in sample selection and pseudo-label optimization.

The authors in [6] investigated the capabilities of BERT-based models in RE tasks. As mentioned in Section 2.2, they ignored the low-resource characteristic. Researchers have explored entropy-based quantitative uncertainty in RE tasks, such as in [15,16,17,18]. However, there are no studies on the RE task in Chinese CTI low-resource scenarios. Moreover, uncertainty propagation in the information extraction stage can lead to cumulative errors in knowledge graph construction, requiring uncertainty control throughout the entire process, from entity recognition and relation extraction to graph fusion [23]. This further highlights the necessity of the research in this paper.

## 3. EDAER Approach

This section first outlines the EDAER approach for extracting entity and relations in Section 3.1. Then, Section 3.2 and Section 3.3 present the detailed EDRMRC and EDRRC models, respectively.

### 3.1. Overview

**Figure 2:**
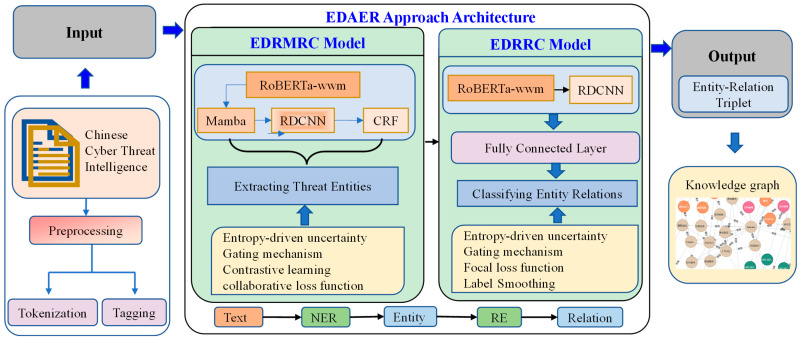
EDAER approach architecture.

Figure 2 illustrates the EDAER approach’s architecture, which takes the preprocessed dataset as input and then generates entity–relation triplets. These triplets can be stored and visualized through the graph database Neo4j [24]. The EDAER architecture comprises two models, namely the entropy-driven RoBERTa_wwm-Mamba-RDCNN-CRF (EDRMRC) and entropy-driven RoBERTa_wwm-RDCNN (EDRRC) models. EDRMRC can identify threat entities from unstructured text. With the extracted entities, EDRRC can classify the logical relationships between these entity pairs. EDRMRC uses RoBERTa_wwm to capture the global semantic meaning of Chinese words and the Mamba module to handle long sequences efficiently with a lower computational cost, and it utilizes RDCNN to capture local features. To ensure that the output makes sense, a CRF layer is employed at the end to verify the validity of the predicted label sequences.

The identified entities are then used as input to the EDRRC model along with their context. The RE stage of extracting relations aims only to categorize the relationship type rather than labeling a sequence. Thus, the EDRRC model uses a more concise architecture without a CRF layer. Instead, it utilizes RDCNN to capture local features relevant to the relationships and uses a fully connected layer to produce the final classification result.

The core novelty that distinguishes EDAER from the existing approaches is the application of the entropy-based uncertainty measurement technique in EDRMRC and EDRRC. In low-resource scenarios, existing models cannot reliably assess the prediction confidence.

Our approach, EDAER, addresses this weakness by using entropy to dynamically quantify the prediction confidence. A high entropy value indicates that the model is uncertain about its decision. The EDRMRC model combines uncertainty measurement with contrastive learning, enabling effective distinguishment between reliable and unreliable samples. Therefore, the EDRMRC model can work well in low-resource environments and significantly improves the model’s stability when facing noisy labels.

### 3.2. EDRMRC Model

**Figure 3:**
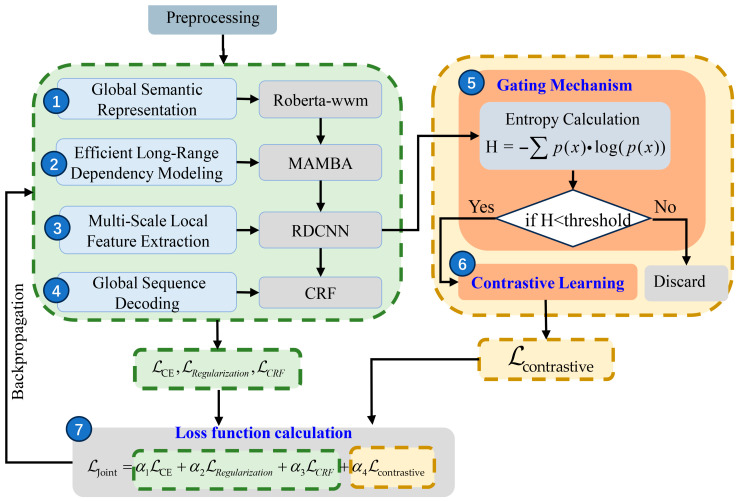
EDRMRC model architecture for NER task.

Figure 3 illustrates the EDRMRC model architecture, which aims to address the inherent complexities of Chinese CTI texts, such as sparse entities and long, unstructured descriptions. The architecture combines a series of collaborative modules to enhance the feature extraction capabilities and develops an adaptive learning strategy according to uncertainty estimation. The following seven components are included in EDRMRC, as detailed in Section 3.2.1, Section 3.2.2, Section 3.2.3, Section 3.2.4, Section 3.2.5, Section 3.2.6 and Section 3.2.7.

(1) Global semantic representation. RoBERTa_wwm [7] is utilized as the foundational pretrained model. It employs a whole-word masking method to reflect fine-grained semantic information, which is critical for understanding domain-specific Chinese terminology.

(2) Long-rang dependency modeling. Mamba [8] is applied to improve the efficiency in processing lengthy reports.

(3) Multi-scale local feature extraction. RDCNN is applied to identify short-distance local features through multi-scale dilated convolution, and then the model’s perception of the local context is improved.

(4) Global sequence decoding. CRF [25] is applied to optimize the global sequence of entity labels, and then the consistency and logical coherence of the final output are assured.

(5) Entropy-based dynamic gating mechanism. It calculates the token-level Shannon entropy to measure the prediction uncertainty. A high entropy value serves as an indicator of low confidence. It also dynamically adjusts the thresholds according to the entropy distribution. In particular, it filters low-confidence samples to optimize the training efficiency.

(6) Uncertainty-guided contrastive learning mechanism. This mechanism treats low-entropy tokens as positive samples and high-entropy tokens as hard negative samples. Based on these samples, it applies a contrastive loss function to improve the model’s discriminative capabilities in low-resource and noisy-label scenarios.

(7) Joint loss function. This involves the weighted summation of the cross-entropy loss, entropy regularization loss, CRF loss, and contrastive loss in order to optimize the generalizability and balance classification accuracy and sequence coherence.

#### 3.2.1. RoBERTa_wwm-Based Global Semantic Representation

EDRMRC uses RoBERTa_wwm [7] as the pretraining model to obtain the semantic representation in the EDRMRC model. The representation incorporates word-level information, making it more suitable for Chinese NER tasks. The performance of an NER model is heavily influenced by the pretraining stage. Unlike the standard BERT [7], which relies on character-level masking, RoBERTa_wwm incorporates word-level information, making it more robust for Chinese NER tasks, where word boundaries are critical. We summarize the strengths of RoBERTa_wwm over BERT in terms of the following three key aspects.

(1) The masking scheme. In Chinese, a single character often has multiple meanings, while semantic clarity usually emerges at the word level. RoBERTa_wwm employs a full-word masking strategy. As illustrated in Table 2, when processing a CTI sentence like “BITTER APT组织是一个长期活跃的境外网络攻击组织。”, the standard BERT might only mask the character “组”, which the model can easily predict based on “织”. That is, the masked result is “BITTER APT组织是一个长期活跃的[MASK] [MASK]网络攻击组织。”. In contrast, RoBERTa_wwm masks the entire phrase “组织” (organization), forcing the model to capture the complete semantic feature of the technical term. Then the masked result under RoBERTa_wwm is “BITTER APT组织是一个长期活跃的[MASK][MASK][MASK][MASK][MASK][MASK][MASK] [MASK]。”. This is particularly advantageous for identifying domain-specific terminology in cybersecurity reports.

(2) The pretraining task. To enhance the robustness of contextual representations, RoBERTa_wwm replaces the static masking of BERT with dynamic masking. Instead of using a fixed mask throughout the pretraining phase, the masked tokens are reselected in each training iteration. This allows the model to observe different masking patterns for the same sentence, preventing overfitting on specific patterns and improving its ability to generalize to diverse and unstructured CTI texts.

(3) Next sentence prediction (NSP) tasks in the pretraining phase. RoBERTa_wwm removes the NSP task used in BERT. Research has shown that the NSP task can sometimes degrade performance on downstream tasks because it overly simplifies the learning objective. By focusing solely on the improved masked language modeling (MLM) objective over larger corpora, the model can learn deeper semantic dependencies, which are essential for recognizing nested or complex entities in the Chinese CTI domain.

Upon completion of pretraining, the model is integrated into the RMRC framework and adapted to specialized threat intelligence data through fine-tuning.

#### 3.2.2. Mamba-Based Efficient Long-Range Dependency Modeling

Mamba [8] is selected as a lightweight and efficient model for sequence modeling tasks. It leverages a selective state-space mechanism (SSM) to enhance its context awareness. Without applying fixed dynamics to all inputs, Mamba dynamically adjusts its state-space parameters (A, B, and C) based on the input sequence. This selective mechanism allows Mamba to focus on relevant parts of the sequence, akin to attention mechanisms but with a significantly lower computational cost. Formally, the selective SSM can be expressed as in Equations (1) and (2):

(1) $$h_{t}=A_{t}h_{t-1}+B_{t}x_{t}$$

(2) $$y_{t}=C_{t}h_{t}$$

where $A_{t}$, $B_{t}$, and $C_{t}$ are input-dependent matrices computed at each time step t. This adaptability makes Mamba particularly effective for tasks like Chinese NER, where contextual cues at the word or phrase level are critical for accurate entity tagging.

In the context of Chinese NER, Mamba serves as an efficient encoder to generate contextualized representations of input sequences. After pretraining on a large-scale Chinese corpus (e.g., Chinese Wikipedia), Mamba can be fine-tuned for downstream tasks by appending a task-specific layer, such as a fully connected layer or a CRF decoding layer. The linear complexity of Mamba allows for faster training and inference compared to Transformer-based models, making it a practical choice for resource-constrained environments or applications requiring real-time processing.

#### 3.2.3. RDCNN-Based Multi-Scale Local Feature Extraction

RDCNN is used as the encoder due to its capacity to capture broader contextual information, which is achieved through its dilated convolution mechanism. The two key features of the RDCNN architecture are outlined below.

(1) Dilated Convolutional Neural Network

Equation (3) denotes the convolution operation in the convolutional layer of the dilated CNN:

(3) $$z_{k}=\sum_{m=-p}^{p}g_{m}\cdot y_{k+m\cdot e}+c$$

where k indicates the word’s position within the input sentence, y represents the input sentence, $y_{k}$ is the $k_{th}$ word in the input sentence, 2p + 1 is the window size, g is the filter function, m is the relative position of offset k, $g_{m}$ is the filter parameter at offset m, and e>1 is the expansion coefficient of the dilated CNN. When e=1, the dilated convolution becomes equivalent to a standard convolution operation [26].

As shown in Equation (4), $d_{k}$ is the information obtained after further functional deformation of the information extracted from the convolution, where W is the convolution kernel weight and b is the bias:

(4) $$d_{k}=\mathrm{relu}(Wz_{k}+b)$$

This yields a feature map, as shown in Equation (5):

(5) $$D=(d_{1},d_{2},\ldots ,d_{n})$$

(2) Residual Connection

The straightforward stacking of dilated convolutional layers may lead to network degradation during training. To mitigate this issue, residual connections [27] are incorporated, as expressed in Equation (6). Within each residual block, a branch applies a transformation residual function F(y) to the input x, and the result is added to the original input:

(6) o=y+F(y)

**Figure 4:**
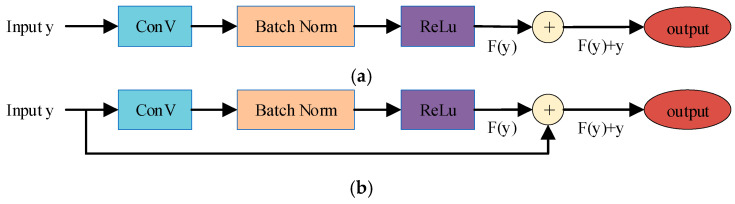
Comparison between (**a**) a standard convolutional layer and (**b**) the proposed residual block.

Residual connections enable the direct propagation of original information to deeper network layers—a design that has been repeatedly demonstrated to enhance the training stability and representational capacity of deep neural networks [27,28]. Figure 4a depicts a standard convolutional layer without residual connections, and Figure 4b presents the residual block adopted in our EDRMRC approach. Our residual block consists of a dilated convolutional block (ConV), a normalization layer (Batch Norm), and a nonlinear activation layer (ReLu). Note that EDRMRC employs BatchNorm [29] to accelerate convergence and improve the training stability, and it uses a rectified linear unit [30] as the activation function.

#### 3.2.4. CRF-Based Global Sequence Decoding

EDRMRC employs CRF as the decoding module to determine the globally optimal sequence of named entity labels. By modeling transition constraints between adjacent tags, the CRF layer enhances the coherence of predictions and prevents the occurrence of invalid tag sequences.

CRF computes two types of scores: (1) emission scores, which reflect the compatibility between tokens and labels, and (2) transition scores, which capture dependencies between consecutive labels. Its objective function is designed to evaluate the quality of a label sequence by combining these two types of scores.

Let $x=(w_{1},w_{2},\ldots ,w_{N})$ be an input sequence of length N, and let $y=(y_{1},y_{2},\ldots ,y_{N})$ denote a candidate label sequence. The emission score is derived from the feature representation of each token, typically produced by a neural encoder (RDCNN), and Equation (7) is used to calculate it:

(7) $$E(x,y)=\sum_{i=1}^{N}\phi (w_{i},y_{i})$$

where $\phi (w_{i},y_{i})$ represents the score in assigning label $y_{i}$ to token $w_{i}$.

The transition score is modeled by a parameterized matrix that quantifies the likelihood of label-to-label transitions, as shown in Equation (8):

(8) $$T(y)=\sum_{i=1}^{N-1}\psi (y_{i},y_{i+1})$$

where $\psi (y_{i},y_{i+1})$ denotes the transition score from label $y_{i}$ to $y_{i+1}$.

The total sequence score is then given in Equation (9):

(9) S(x,y)=E(x,y)+T(y)

Given this definition, the probability of a label sequence is normalized over all possible sequences, as shown in Equation (10):

(10) $$p(y\left|x\right.)=\frac{\exp(S(x,y))}{\sum_{\tilde{y}\in Y(x)}\exp(S(x,\tilde{y}))}$$

where Y(x) denotes the set of all possible label sequences for input x.

The negative log-likelihood loss is defined in Equation (11):

(11) $$L(x,y^{*})=-\log p(y^{*}|x)=-S(x,y^{*})+\log\underset{\tilde{y}\in Y(x)}{\sum }\exp(S(x,\tilde{y}))$$

where $y^{*}$ is the ground-truth label sequence. During inference, the optimal label sequence is obtained by maximizing the score function, as given in Equation (12):

(12) $$y^{o}=\arg\underset{y\in Y(x)}{\max}S(x,y)$$

#### 3.2.5. Entropy-Based Dynamic Gating Mechanism

Low-confidence predictions can affect model performance in low-resource scenarios. EDRMRC solves this issue via introducing an entropy-based dynamic gating mechanism. Note that entropy is an effective metric to measure the uncertainty of a probability distribution. Our mechanism first utilizes entropy to measure the uncertainty of the model’s predictions. Then, the entropy is compared with a defined threshold (Threshold) to exclude low-confidence samples. If the entropy exceeds Threshold, the sample is considered of low confidence and is then removed from training. Therefore, training can be optimized, and the model’s robustness and accuracy can be improved.

We define $x_{t}$ to denote the input sample, $P(x_{t})$ to denote the predicted probability distribution, and $H(x_{t})$ to denote the uncertainty of predicting $x_{t}$. Then, Equation (13) can be used to calculate entropy $H(x_{t})$:

(13) $$H(x_{t})\;=\;-\sum p(x_{t})\cdot \log(p(x_{t}))$$

The higher the entropy $H(x_{t})$, the more uncertain the model is about its prediction. To effectively filter out low-confidence samples during training, we employ a dynamic entropy threshold mechanism. The dynamic entropy threshold is computed using Equation (14):

(14) $${\mathrm{Threshold}}_{t}=\lambda \cdot H(x_{t})+\beta$$

where λ and β are adjustable hyperparameters that control the influence of entropy on the threshold. The threshold is dynamically adjusted based on the entropy distribution observed during the training process, allowing for the flexible filtering of low-confidence predictions. To determine their optimal values, we conducted a grid search. The optimal combination (λ = 0.3, β = 0.1) was selected to minimize the validation loss, ensuring the robustness of the dynamic threshold mechanism.

During training, low-confidence samples (with high entropy) are dynamically filtered out. Specifically, when the entropy of a prediction exceeds the threshold, the sample is marked as low-confidence and excluded from training. This mechanism is implemented as in Equation (15):

(15) $$\mathrm{LowCS}(x_{t})=\left\{\begin{array}{ll} \mathrm{True} & \mathrm{if}\;H(x_{t})\geq {\mathrm{Threshold}}_{t}\\ \mathrm{False} & \mathrm{if}\;H(x_{t})<{\mathrm{Threshold}}_{t} \end{array}\right.$$

where $\mathrm{LowCS}(x_{t})$ denotes the low-confidence sample flag for the t-th prediction $x_{t}$.

Only high-confidence samples—those with entropy below the dynamic threshold—are used for training. This mechanism effectively avoids the negative impact of low-confidence samples on the model’s training process, improving the learning efficiency and prediction accuracy.

By introducing the entropy-based gating mechanism, there is an improvement in the model’s performance in low-resource environments, particularly when dealing with highly uncertain samples.

#### 3.2.6. Uncertainty-Guided Contrastive Learning Mechanism

Traditional learning methods like SVM often rely on a large number of labeled samples and thus cannot work well in low-resource environments where labeled data is scarce. Contrastive learning provides an efficient way to learn meaningful features by maximizing the similarity between positive samples and minimizing the similarity between negative samples. This helps the EDRMRC model to learn discriminative features that distinguish between different samples.

EDRMRC uses high-confidence samples generated from entropy-based dynamic gating as input to the contrastive learning mechanism, while low-confidence (high-entropy) samples are filtered out and excluded from the contrastive learning computation. The positive and negative samples for contrastive learning are divided based on their true annotation labels. Samples with the same label as the anchor sample are regarded as positive samples, and those with different labels are considered negative samples.

The contrastive loss function is defined as in Equation (16):

(16) $$L_{contrastive}=-\log\frac{\exp(sim(z_{i},z_{j})/\tau )}{\sum_{k=1}^{N}\exp(sim(z_{i},z_{k})/\tau )}$$

where $sim(z_{i},z_{j})$ represents the similarity between the embeddings of samples $x_{i}$ and $x_{j}$, and τ is a temperature parameter that controls the scaling of the similarities. By minimizing this contrastive loss, the EDRMRC model learns to bring the same labels closer together in the feature space, while pushing different labels further apart.

In practice, contrastive learning helps to improve the model’s generalizability by reducing the impact of low-confidence samples during training. This enhances the EDRMRC model’s robustness, particularly in low-resource environments where labeled data is limited.

#### 3.2.7. Joint Loss Function for EDRMRC Training

This loss function effectively integrates the losses of different tasks by adjusting the weights between tasks, ensuring that each task’s learning goal is adequately optimized. The joint loss function in the EDRMRC model includes the following components: cross-entropy loss, entropy regularization loss, CRF loss, and contrastive loss.

(1) Cross-Entropy Loss

This is used for classification tasks to measure the difference between the predicted label distribution and the true label distribution. For a given input x and the true label y, Equation (17) defines the formula for the cross-entropy loss $L_{\mathrm{CE}}$:

(17) $$L_{\mathrm{CE}}=-\sum_{i=1}^{N}y_{i}^{*}\log{\hat{y}}_{i}$$

where $y_{i}^{*}$ is the true label and ${\hat{y}}_{i}$ is the predicted probability distribution.

(2) Entropy Regularization Loss

This is used to control the output distribution of the model, making it smoother and preventing overfitting. Equation (18) defines the formula for entropy regularization loss $L_{\mathrm{Entropy}}$:

(18) $$L_{\mathrm{Entropy}}=\sum_{i=1}^{N}H({\hat{y}}_{i})$$

where $H({\hat{y}}_{i})$ is the entropy of the predicted probability distribution ${\hat{y}}_{i}$.

(3) CRF Loss

For sequence labeling tasks, the CRF loss optimizes the coherence of the label sequence. Equation (19) defines the formula for CRF loss $L_{\mathrm{CRF}}$:

(19) $$L_{\mathrm{CRF}}=-\log\frac{\exp(\mathrm{score}(Y^{*},X))}{\sum_{Y}\exp(\mathrm{score}(Y,X))}$$

where $\mathrm{score}(Y^{*},X)$ is the score of the true label sequence $Y^{*}$ and input X, and the denominator is the sum of the scores of all possible label sequences.

(4) Contrastive Loss

In low-resource environments where labeled data is scarce, traditional learning methods often rely on a large number of labeled samples. Contrastive learning provides an efficient way to learn meaningful features by maximizing the similarity between positive samples and minimizing the similarity between negative samples. This helps the model to learn discriminative features that distinguish between different samples. The core of contrastive learning lies in the contrastive loss function, which operates by calculating the similarity between positive and negative samples. It encourages the model to pull positive samples closer together and push negative samples further apart. Specifically, for each pair of positive and negative samples, the model minimizes the similarity between positive samples and maximizes the similarity between negative samples. Equation (2) defines the formula for contrastive loss $L_{\mathrm{contrastive}}$:

(20) $$L_{\mathrm{contrastive}}=-\log\frac{\exp(\mathrm{sim}(z_{i},z_{j})/\tau )}{\sum_{k=1}^{N}\exp(\mathrm{sim}(z_{i},z_{k})/\tau )}$$

where $\mathrm{sim}(z_{i},z_{j})$ represents the similarity between the embeddings of samples $x_{i}$ and $x_{j}$, and τ is the temperature parameter used to control the scaling of similarities.

The final joint loss function is obtained by weighting and summing the losses of all components mentioned above. Equation (21) defines the formula for computing the total loss function:

(21) $$L_{t}=\alpha_{1}L_{\mathrm{CE}}+\alpha_{2}L_{\mathrm{Entropy}}+\alpha_{3}L_{\mathrm{CRF}}+\alpha_{4}L_{\mathrm{contrastive}}$$

where $\alpha_{1},\alpha_{2},\alpha_{3},\alpha_{4}$ are the weighting coefficients for each component of the loss function, adjusting the contribution of each loss term.

### 3.3. Entropy-Driven Relation Extraction Model (EDRRC)

**Figure 5:**
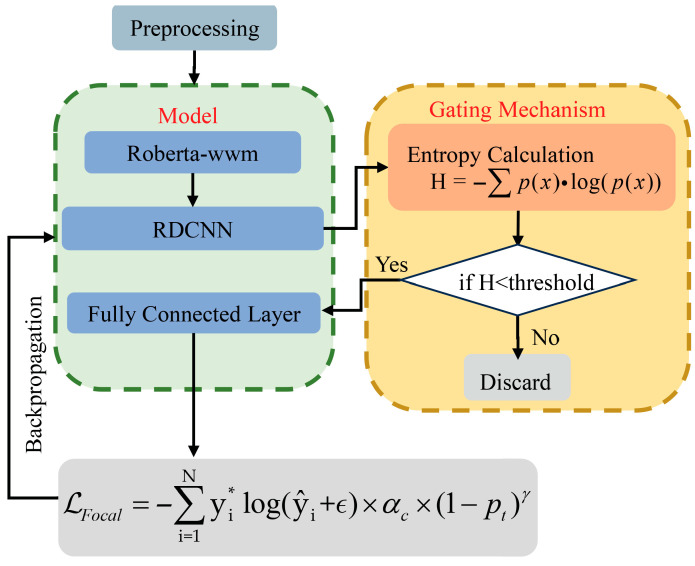
EDRRC model architecture for RE task.

For the RE task, we propose the entropy-driven RoBERTa_wwm-RDCNN (EDRRC) model. Figure 5 illustrates the EDRRC model architecture. This architecture combines the RoBERTa_wwm encoder with a CNN to classify the relationships between entity pairs. A major challenge in this task is class imbalance, which often leads to inaccurate predictions for minority classes. EDRRC applies the entropy-based dynamic gating mechanism proposed in Section 3.2.7 and combines it with the focal loss and label smoothing techniques to enhance the model’s performance on imbalanced data.

The entropy-based dynamic gating mechanism accurately quantifies the uncertainty of model predictions by calculating the entropy of the probability distribution of relation predictions (the calculation method is the same as the entropy formula in Section 3.2.5). On this basis, the model adopts a dynamic threshold (refer to Section 3.2.5), filtering out high-entropy, low-confidence samples to avoid the interference of noisy labels in the training process.

Equation (22) gives the formula for calculating the focal loss, which based on the standard cross-entropy loss (CE loss). It aims to reduce the impact of easy samples and forces the model to focus more on hard-to-classify samples during training.

(22) $$L=\alpha_{c}\times {\left(1-p_{t}\right)}^{\gamma }\times \mathrm{CE}\_\mathrm{loss}$$

where $\alpha_{c}$ is the weight for class c, used to adjust the loss impact for different classes; $p_{t}$ is the predicted probability for the true class; and γ is the focusing parameter, typically set to 2.0, which increases the attention paid to difficult samples. In the RE task, different relationship categories may have varying importance and frequencies. To optimize the model’s focus, different $\alpha_{c}$ values are set for each class.

Label smoothing is a regularization technique used to reduce overfitting and enhance the model’s generalizability. By smoothing hard labels into soft labels, label smoothing reduces the model’s dependency on specific samples from the training data. For example, the hard label [0, 0, 1, 0] is smoothed into [0.016, 0.016, 0.95, 0.016]. The formula for label smoothing is

(23) $$\mathrm{CE}\_\mathrm{loss}\_\mathrm{smooth}=-\sum_{i=1}^{N}y_{i}^{*}\log({\hat{y}}_{i}+\epsilon )$$

where ϵ is the smoothing coefficient, typically set to 0.05; $y_{i}^{*}$ is the true label; and ${\hat{y}}_{i}$ is the predicted probability.

By combining the focal loss and label smoothing, the final RE loss function can be expressed as

(24) $$L=\alpha_{c}\times {\left(1-p_{t}\right)}^{\gamma }\times \mathrm{CE}\_\mathrm{loss}\_\mathrm{smooth}$$

where $\alpha_{c}$ is the weight for class c, $p_{t}$ is the predicted probability for the true class, γ is the focusing parameter (typically set to 2.0), and CE_loss_smooth is the cross-entropy loss with label smoothing.

This combination enables the focal loss to alleviate the class imbalance issue in RE by paying more attention to difficult-to-classify samples. In addition, the model’s generalizability can be improved and overfitting prevented via label smoothing.

Thus, the performance of the model in RE tasks is improved, particularly in cases of class imbalance and challenging samples.

## 4. Establishment of MyCDTier Dataset

### 4.1. Data Acquisition, Preprocessing, and Annotation

We collect data from open-source cybersecurity reports published by authoritative platforms, including Tencent Security [31], Alibaba Cloud Security [32], 360 Security [33], ThreatBook [34], FreeBuf [35], and Qianxin [36]. These reports describe rich unstructured textual information about APT attacks, vulnerabilities, and malware campaigns. We preprocess the data via cleaning them to remove html tags, garbled text, and irrelevant metadata. There are two specialized subsets in the obtained dataset: a threat entity extraction dataset containing 7861 sentences and an entity–relation extraction dataset comprising 3918 sentences.

Named entity recognition (NER) adopts the BIO tagging method, and relation extraction (RE) uses a unified annotation format consisting of “type number + head entity (h) + relation + tail entity (t)”. The overall annotation process is divided into five stages: tentative annotation → consensus annotation → result validation → full corpus annotation → cross-validation.

### 4.2. Named Entity Types

We consider 16 distinct entity types defined in our ontology, which are Admin, Device, Hardware, Software, Operating System, Service, Network, IP, Port, Protector, Countermeasure, Adversary, Attack Vector, Vulnerability, Malware, and Worm, as defined in Table 3.

These 16 entity types are classified into four categories.

(1) The first is the Asset category, which represents the fundamental environment of the attack target. There are several type entities in this category: Admin, Device, Operating System, Hardware, and Software. They reflect the physical and logical infrastructures of the target systems.

(2) The second is the Feature category, which describes the basic attributes of these targets. It includes IP, Port, Service, and Network entities. They describe the unique identifiers and service characteristics of network nodes.

(3) The third is the Attack category, which includes entities capable of launching attacks against specific targets. There are five type entities in this category: Adversary, Attack Vector, Vulnerability, Malware, and Worm. These entities detail the potential sources of threats and the specific means employed to compromise systems.

(4) The fourth is the Protector category, denoting the entities for implementing protective measures. There are two type entities in this category: Protector and Countermeasure. These correspond, respectively, to the individuals or teams executing defense strategies and the specific technical measures adopted to safeguard the system, such as access control protocols and security audit mechanisms.

### 4.3. Relation Types

**Figure 6:**
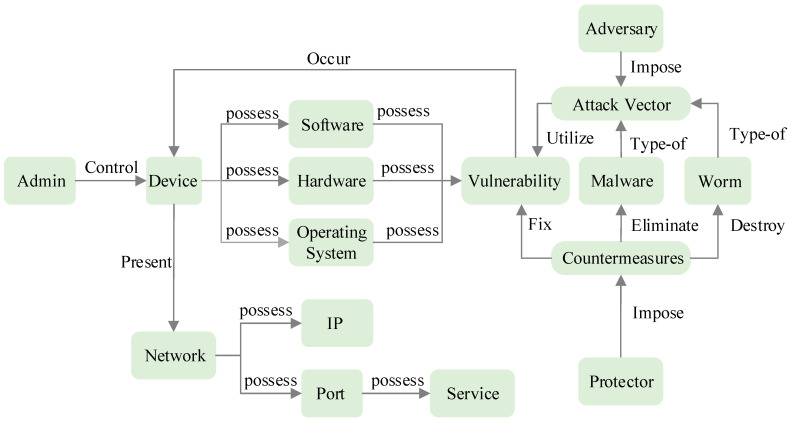
The 9 relationship types for the 16 key knowledge objects.

We selected 3918 sentences and annotated them based on the nine relationship categories defined in our ontology: Control, Occur, Possess, Present, Utilize, Impose, Type-of, Eliminate, and Destroy. The annotated dataset was randomly partitioned into training, testing, and validation sets with proportions of 70%, 20%, and 10%, respectively. Figure 6 provides a comprehensive illustration of these nine relationship types and illustrates how they connect the sixteen key knowledge entities (defined) across the four categories. The relationship types are explained in the following.

The relationships in the dataset capture both internal interactions within categories and complex cross-category associations. Within specific categories, the logic is hierarchical; for example, in the Asset category, admins control devices, while devices possess components like software, hardware, and operating systems. Similarly, in the Feature category, a network possesses IPs and ports. The Adversary category involves dynamic interactions where adversaries impose specific vectors, and these vectors utilize vulnerabilities. Malware and worms are categorized as specific instances of type-of attack vectors. In the Protector category, protectors impose countermeasures to execute protection strategies.

Crucially, the dataset also defines interactions that span across different categories. For instance, a device in the Asset category can be presented through a network in the Feature category. Devices and their underlying components (software, hardware, operating systems) are linked to vulnerabilities in the Adversary category through occur or possess relationships. Furthermore, countermeasures in the Protector category directly counter specific threats; they fix vulnerabilities, eliminate malware, and destroy viruses.

## 5. Experimental Evaluation

### 5.1. Experimental Setup

#### 5.1.1. Experimental Environment

All experiments were conducted in a Linux 6.5.0-45-generic operating system environment, using the Python 3.12 programming language and PyTorch 2.0.1 deep learning framework. We utilized an NVIDIA GPU with CUDA support to accelerate computation and configured CUDA debugging environment variables to improve the computational stability. RoBERTa_wwm was used as the pretrained language model, configured with a maximum sequence length of 128 tokens and batch size of 16, and we used the AdamW optimizer for model training, with an initial learning rate of 1 × 10^−5^ and 50 training epochs. In the model architecture, the Mamba module’s state dimension, convolution dimension, and expansion factor were set to 16, 4, and 2, respectively. The RDCNN module was configured with 256 convolution kernels; kernel sizes of 3, 5, and 7; and dilation rates of 1, 2, and 4. We utilized a CRF layer for sequence labeling, employing the Xavier initialization strategy, and implemented an early stopping mechanism (patience = 30) to prevent overfitting.

#### 5.1.2. Dataset

The dataset was used to validate the effectiveness of the proposed model on Chinese CTI data. Experiments were conducted using the following two datasets: CDTier and MyCDTier.

(1) CDTier Dataset [6]. This is an open-source dataset for Chinese CTI, accessible at https://github.com/MuYu-z/CDTier (accessed on 1 June 2025). For NER, it includes 100 CTI reports, 3744 threat sentences, and 4259 threat knowledge objects. The dataset is annotated with five entity types (Attacker, Campaign, Industry, Region, and Tools) and their beginning (B-) and inside (I-) tags. Thus, there are 10 unique labels. For RE, there are 100 CTI reports, 2598 threat sentences, and 2562 knowledge object relations, covering 11 types of relationships (e.g., utilize, target) based on standards like STIX.
(2) MyCDTier. This is detailed in Section 4. It is a proprietary dataset developed in-house for cybersecurity NER and RE, featuring the 16 entity types defined in Table 3. The RE task involves extracting nine specific relationships between these entity types—namely, Control, Occur, Possess, Present, Utilize, Impose, Type-of, Eliminate, and Destroy—which significantly increases the task complexity.

#### 5.1.3. Evaluation Metrics

We perform an evaluation using the four basic metrics of accuracy, precision, recall, and the F1-score. In addition, we evaluate the precision, recall, and F1-score in two cases: the macro-average and the micro-average. We first define TP to denote true positives (correctly predicted entity labels), TN to denote true negatives (correctly predicted non-entity labels), FP to denote false positives (non-entities incorrectly predicted as entities), and FN to denote false negatives (entities not identified).

(1) Accuracy

Accuracy, defined in Equation (25), quantifies the proportion of correctly predicted labels relative to the total number of labels. Accuracy in Chinese NER is an overall measure of correctness across all labels, involving the prevalent non-entity “O” label. However, due to the dominance of “O” labels in typical NER datasets, accuracy may not completely denote performance for entity-specific predictions:

(25) $$\mathrm{Accuracy}=\left(TP+TN\right)/\left(TP+TN+FP+FN\right)$$

(2) Precision

Precision, defined in Equation (26), measures the proportion of predicted entity labels that are correct. Here, TP means the number of correctly identified entities, and $\left(TP+FP\right)$ denotes the number of predicted entities. High precision is critical in Chinese NER to ensure that predicted entities (e.g., person names, locations) are accurate, minimizing false positives.

(26) $$\mathrm{Precision}=TP/\left(TP+FP\right)$$

(3) Recall

Recall measures the proportion of actual entities that are correctly identified. It is defined as

(27) $$\mathrm{Recall}=\frac{TP}{TP+FN}$$

Here, TP + FN denotes the total number of actual entities. Recall is essential for evaluating the model’s ability to capture all true entities, particularly in Chinese NER, where missing entities can significantly impact downstream applications.

(4) F1-score

The F1-score, defined in Equation (28), represents both precision and recall, resolving the trade-off in imbalanced datasets when entity labels are sparse.

(28) $$F1=\left(2\times TP\right)/\left(2\times TP+FP+FN\right)$$

(5) Macro-average

The macro-average represents the arithmetic mean of metrics across all entity types, regarding each type equally regardless of its frequency. For K entity categories (excluding “O” labels), we define ${\mathrm{Precision}}_{i}=TP_{i}/\left(TP_{i}+FP_{i}\right)$, ${\mathrm{Recall}}_{i}=TP_{i}/\left(TP_{i}+FN_{i}\right)$, and ${F1-\mathrm{score}}_{i}=\left(2\times {\mathrm{Precision}}_{i}\times {\mathrm{Recall}}_{i}\right)/\left({\mathrm{Precision}}_{i}+{\mathrm{Recall}}_{i}\right)$. Then, we obtain the macro-average metrics, as in Equation (29):

(29) $$\begin{array}{l} \mathrm{Macro-Precision}=\sum_{j=1}^{K}{\mathrm{Precision}}_{j}/K\\ \mathrm{Macro-Recall}=\sum_{j=1}^{K}{\mathrm{Recall}}_{j}/K\\ \mathrm{Macro-F1-score}=\sum_{j=1}^{K}{\mathrm{F1-score}}_{j}/K \end{array}$$

In the NER task, macro-averaging can assure equal weighting, which is critical to Chinese datasets with imbalanced category distributions. Thus, the macro-average is helpful for performance evaluation when entity categories are rare.

(6) Micro-average

The micro-average aggregates predictions across all categories to compute overall metrics, emphasizing performance on frequent categories. The total TP, FP, and FN are summed across all categories: $TP=\sum_{i=1}^{N}TP_{i}$, $FP=\sum_{i=1}^{N}FP_{i}$, $FN=\sum_{i=1}^{N}FN_{i}$. The micro-average metrics are as follows:

(30) $$\begin{array}{l} \mathrm{Micro-Precision}=\frac{\sum_{i=1}^{K}TP_{i}}{\sum_{i=1}^{K}(TP_{i}+FP_{i})}\\ \mathrm{Micro-Recall}=\frac{\sum_{i=1}^{K}TP_{i}}{\sum_{i=1}^{K}(TP_{i}+FN_{i})}\\ \mathrm{Micro-F1}=\frac{2\times \mathrm{Micro-Precision}\times \mathrm{Micro-Recall}}{\mathrm{Micro-Precision}+\mathrm{Micro-Recall}} \end{array}$$

Micro-averaging is well suited for imbalanced datasets, as it reflects the overall performance weighted by category frequency, which is common in Chinese NER tasks.

#### 5.1.4. Models and Baselines

For NER, we compare our method against the following baselines:

- RoBERTa_wwm-RDCNN-CRF. Combines RoBERTa_wwm with RDCNN and CRF. We selected this baseline because RoBERTa_wwm is a state-of-the-art pretrained model optimized for Chinese text, and RDCNN is widely used to capture local contextual features. This combination serves as a strong representative of recent Chinese NER models, allowing us to verify the performance gain from introducing the Mamba module in our architecture.
- BERT-Mamba-RDCNN-CRF. Employs the Chinese version of BERT-base as the PLM, paired with Mamba, RDCNN, and CRF. We included this baseline to compare the impacts of different pretrained language models on performance.
- RoBERTa_wwm-TENER-CRF. Combines RoBERTa_wwm with TENER (Transformer-based) and CRF. We chose this baseline because TENER is a classic Transformer-based architecture for sequence labeling, enabling us to demonstrate the advantages of our Mamba + RDCNN hybrid feature extraction over Transformer-only architectures in CTI scenarios.
- BERT-BiLSTM-GRU-CRF: Uses BERT with LSTM and GRU layers, followed by CRF. This is a well-established hybrid architecture that was widely used in early Chinese NER research. By including it, we can highlight the technical advantages of modern components (e.g., Mamba, RDCNN) over traditional recurrent models in the CTI domain.

For RE, we propose RoBERTa_wwm-RDCNN (RRC), which combines RoBERTa_wwm with a convolutional neural network (CNN) for relation classification. It is compared against BERT, a baseline using BERT-base with a simple classification head.

### 5.2. Experimental Results

#### 5.2.1. Named Entity Recognition Results

Accuracy is not suitable for NER because, during the recognition process, even if all entities are identified as non-entities or remain undetected, this metric can still be very high, thus offering little reference value. Therefore, accuracy is not used as a metric in this section.

(1) Results for CDTier dataset

Table 4 presents the NER performance on the CDTier dataset in terms of the macro-average and micro-average metrics. EDRMRC achieved a macro-average F1-score of 91.72% and a micro-average F1-score of 91.76%, significantly outperforming all baselines in both the macro-average and micro-average F1-scores. This showcases EDRMRC’s ability to effectively recognize entities across both high-frequency and low-frequency categories—namely, in low-resource scenarios. The better performance is mainly due to the integration of the entropy loss and contrastive learning, which allow EDRMRC to better handle noisy labels and enhance the recognition of low-frequency entities.

In comparison, the RMRC method, without the additional entropy loss and contrastive learning mechanisms, achieved a macro-average F1-score of 90.29% and a micro-average F1-score of 90.05%. While satisfactory, RMRC did not outperform EDRMRC due to the absence of the noise mitigation strategies that EDRMRC benefits from. The RoBERTa_wwm-RDCNN-CRF approach works well, with a macro-average F1-score of 88.40% and a micro-average F1-score of 88.19%, but still falls behind RMRC and EDRMRC due to its lack of entropy loss and contrastive learning enhancements. The BERT-Mamba-RDCNN-CRF, RoBERTa_wwm-TENER-CRF, and BERT-BiLSTM-GRU-CRF models demonstrate significantly inferior performance. Table 4 shows that BERT-Mamba-RDCNN-CRF produces a macro-average F1-score of 82.21% and a micro-average F1-score of 84.16%. The macro-average F1-score and micro-average F1-score of RoBERTa_wwm-TENER-CRF are 84.87% and 85.27%, respectively. BERT-BiLSTM-GRU-CRF performs the most poorly. These results suggest the weakness of LSTM/GRU techniques, particularly in dealing with long-range dependencies and complex entity relationships.

**Figure 7:**
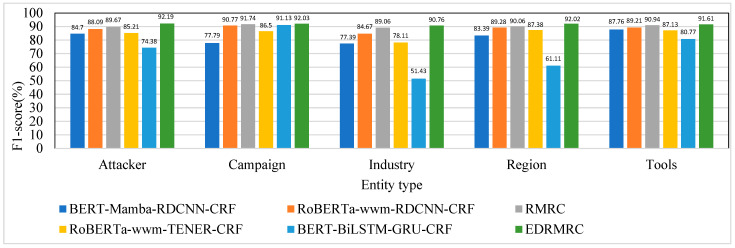
F1-scores of each model on different entities under CDTier dataset.

**Figure 8:**
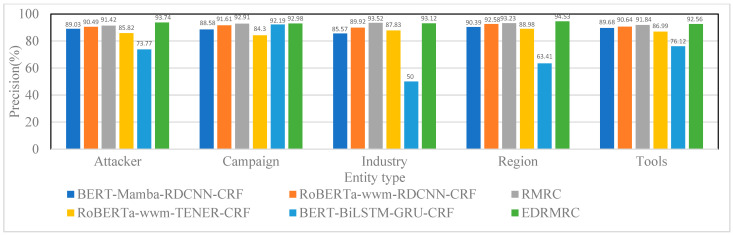
Precision of various models on different entities under CDTier dataset.

**Figure 9:**
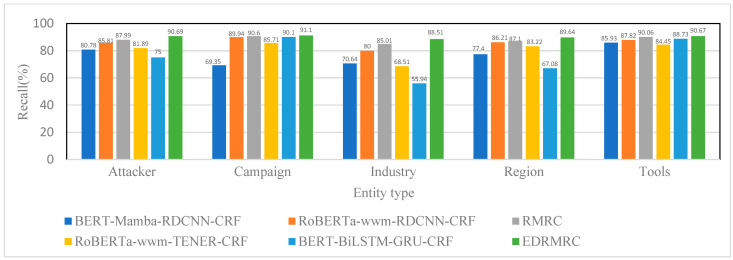
Recall of each model on different entities under CDTier dataset.

The CDTier dataset is annotated with five entity types. Figure 7, Figure 8 and Figure 9 present the results in terms of the F1-scores, precision, and recall for the CDTier dataset, respectively. EDRMRC achieved strong performance across all entity categories: it recorded F1-scores of 92.19% for Attacker, 92.03% for Campaign, 90.76% for Industry, 92.02% for Region, and 91.61% for Tools. In terms of precision, it reached 93.74% for Attacker and 94.53% for Region, while its recall for these entities stayed above 88.5%. This demonstrates its ability to effectively recognize critical entities in these categories. Even for Tools, it maintained strong performance, with precision of 92.56% and recall of 90.67%, with only a small gap compared to other entities. This indicates that EDRMRC excels in recognizing the most critical entities.

In comparison, RMRC without the additional entropy loss and contrastive learning mechanisms still demonstrated strong performance. It achieved F1-scores of 89.67% for Attacker, 91.74% for Campaign, and 89.06% for Industry, with precision of 91.42% for Attacker and 93.52% for Industry, as well as recall of 90.6% for Campaign. While RMRC also performed well, it was outperformed by EDRMRC, especially in low-frequency entity recognition and in handling label noise.

RoBERTa_wwm-RDCNN-CRF performed well but underperformed in comparison with RMRC and EDRMRC. Its F1-score for Attacker was 88.09%, and its precision for Campaign reached 91.61%, resulting in an F1-score of 90.77% for Campaign. However, for other entities, like Industry and Tools, RoBERTa_wwm-RDCNN-CRF’s performance was less impressive, with F1-scores of 84.67% for Industry and 89.21% for Tools.

BERT-Mamba-RDCNN-CRF showed strong performance for Attacker and Tools (with F1-scores of 84.7% and 87.76%, respectively) but struggled with Campaign and Industry entities, achieving relatively low F1-scores of 77.79% for Campaign and 77.39% for Industry. BERT-BiLSTM-GRU-CRF, on the other hand, had the worst performance, especially in recognizing Industry and Region, with F1-scores of 51.43% and 61.11%, respectively, highlighting the limitations of traditional LSTM/GRU architectures in complex NER tasks.

Overall, EDRMRC outperformed all other baselines in recognizing critical cybersecurity entities, showing exceptional precision and recall. The integration of entropy loss and contrastive learning contributed significantly to the superior performance in noisy environments, handling low-frequency entities and maintaining high accuracy across various entity types.

(2) Results for MyCDTier dataset

Table 5 shows the NER performance on the MyCDTier dataset, with EDRMRC emerging as the top performer, followed by RMRC and RoBERTa_wwm-RDCNN-CRF. Regarding the macro-average metrics, EDRMRC achieved a macro-F1-score of 77.49%, while that of RMRC reached 71.92%, and RoBERTa_wwm-RDCNN-CRF scored 63.66%. In terms of the micro-average results, EDRMRC still ranked first, with a micro-F1-score of 75.81%, while RMRC and RoBERTa_wwm-RDCNN-CRF obtained 71.21% and 68.97%, respectively, reflecting their robustness despite the dataset’s complexity.

#### 5.2.2. Relation Extraction Results

Table 6 presents the macro-average and micro-average performance on the CDTier dataset for relation extraction (RE). EDRRC demonstrated significant performance improvements, especially in the macro-average and micro-average F1-scores, as well as the overall recall. EDRRC achieved a macro-average F1-score of 90.84% and a micro-average F1-score of 92.40%, with overall recall of 92.26%. This indicates that, for EDRRC, incorporating the entropy loss improved its robustness in handling label noise and low-confidence predictions, particularly excelling in complex relation extraction tasks.

In comparison, RRC (RoBERTa-RDCNN model) also performed well but with lower results. Its macro-average precision, recall, and F1-score were 77.04%, 72.54%, and 72.90%, respectively, and its micro-average precision, recall, and F1-score were 89.28%, 89.23%, and 88.98%, respectively, which were significantly lower than those of EDRRC. This suggests that, while RRC, based on RoBERTa and RDCNN, has strong contextual modeling capabilities, the absence of the entropy loss mechanism makes it less effective in handling label noise and low-confidence predictions, thus limiting its performance.

The BERT-based method performed the worst, with macro-average precision of 51.00%, macro-average recall of 56.00%, and a macro-average F1-score of 48.00%, as well as micro-average precision of 62.00%, micro-average recall of 60.00%, and a micro-average F1-score of 59.00%. This indicates that, while the BERT-based method excels as a pretrained model, it still faces significant challenges in relation extraction tasks, particularly in handling long-range dependencies and complex relations.

Overall, EDRRC, through the incorporation of the entropy loss and optimization of label noise handling, shows significantly improved relation extraction performance, particularly in low-confidence predictions and handling low-frequency relations. In contrast, the RRC and BERT-based methods performed less effectively in handling label noise and complex relations, demonstrating the effectiveness of adding entropy loss in improving model robustness.

Table 7 reports the label-level RE performance of EDRRC, RRC, and BERT for each relation type on the CDTier dataset. EDRRC demonstrates excellent performance across most relation types, attaining a perfect F1-score of 100.00% on cooperate_with, consist_of, and operating_in and achieving high F1-scores of 94.74%, 96.00%, and 92.50% on alias_of, related_to, and target_at, respectively. Its lowest F1-score, however, is only 75.00% on the launch relation, which is mainly due to contextual ambiguity (its semantic boundaries overlap with use) and the scarce training samples in the dataset, making it challenging for the model to capture the inherent semantic features of this relation. For RRC, it performs well on high-support relations such as target_at (94.68%) and operating_in (95.51%), yet it faces severe challenges with rare instances (e.g., develop, 51.61%). BERT’s performance is suboptimal overall, with a peak F1-score of 68.00% on target_at; notably, it achieves 100% recall on kin but only an 18.00% F1-score due to extremely low precision (10.00%), and it records a very low F1-score of 25.00% on consist_of.

Consistent with the micro-average results in Table 6, Table 8 further verifies the superiority of EDRRC, which achieves the highest overall accuracy of 92.26% on the CDTier dataset, followed by RRC (89.23%) and BERT (60.00%). On the MyCDTier dataset, EDRRC achieves the leading accuracy of 94.23%, far exceeding that of RRC (92.26%) and the baseline BERT (82.95%), with a 15.28% improvement over BERT. This outstanding performance verifies that EDRRC can effectively capture contextual and relational features, owing to the whole-word masking strategy and residual CNN module for modeling local dependencies.

In conclusion, the results prove the strong robustness and cross-dataset adaptability of EDRRC, validating its excellent performance in domain-specific relation extraction tasks beyond the CDTier dataset. The BERT-based method’s inferior performance reflects its insufficiency in processing complex domain-specific relations in MyCDTier, which stems from its token-level masking and limited feature extraction abilities. The evident performance gap demonstrates that MyCDTier demands deeper semantic understanding of relational and linguistic patterns, where EDRRC shows distinct advantages.

## 6. Conclusions and Future Work

This paper advances Chinese cyber threat intelligence (CTI) in low-resource scenarios by proposing two models for extracting named entities and their corresponding relations. Additionally, we construct a Chinese CTI dataset that encompasses a broader range of entity and relation types. Experimental results obtained on both the public CDTier dataset and our self-built dataset demonstrate that our proposed models outperform existing baselines. We summarize the findings from the experiments as follows:

(1) In both NER and RE tasks, the PLM model RoBERTa_wwm significantly outperforms BERT.
(2) In NER tasks, Mamba achieves superior results compared to other methods.
(3) In both NER and RE tasks, the entropy-based dynamic gating mechanism is helpful for performance improvement.
(4) In NER tasks, the uncertainty-guided contrastive learning mechanism is helpful for performance improvement.

Nonetheless, several challenges remain unaddressed. Future research can be directed along the following four avenues: (1) expanding the coverage of Chinese CTI sources to enrich the dataset’s perspectives, (2) incorporating diverse CTI sources to enhance the dataset’s quality and robustness, (3) leveraging the identified entities and relations to detect threats and inform the design of defense mechanisms, and (4) investigating how to place the experimental results within the context of theory building.

## Figures and Tables

**Table 1:** Comparison of models for NER and RE tasks.

Ref.	English CTI Dataset	Chinese CTI Dataset	NER	RE	Joint/Pipeline	Entropy-Based Uncertainty Measurement	Contrastive Learning
[11] 2022	✗	✗	✓	✗	-	✓	✗
[12] 2020	✓	✓	✓	✗	-	✓	✗
[9] 2025	✓	✗	✓	✗	-	✓	✗
[10] 2025	✓	✗	✓	✗	-	✓	✗
[13] 2024	✗	✗	✓	✗	-	✓	✓
[14] 2025	✗	✗	✓	✗	-	✓	✓
[15] 2024	✗	✗	✗	✓	-	✓	✗
[16] 2023	✗	✗	✗	✓	-	✓	✗
[17] 2025	✗	✗	✗	✓	-	✓	✗
[18] 2023	✗	✗	✓	✓	Joint	✓	✗
[4] 2025	✗	✗	✓	✓	Pipeline	✗	✗
[5] 2023	✗	✓	✓	✗	-	✗	✗
[6] 2023	✗	✓	✓	✓	Pipeline	✗	✗
Ours	✗	✓	✓	✓	Pipeline	✓	✓

Regarding English/Chinese CTI datasets, ✗ means that the work is not applicable to English/Chinese CTI datasets, and ✓ means that the work is applicable to English/Chinese CTI datasets. Regarding NER(RE), ✗ means that the work does not consider NER(RE) tasks, and ✓ means that the work considers NER(RE) tasks. For Joint/Pipeline, ‘Joint/Pipeline’ means that the work considers NER and RE tasks simultaneously or in a pipeline. ‘-’ means that the work only considers NER or RE tasks. In terms of entropy-based uncertainty measurement, ✗ means that the work does not apply entropy, and ✓ means that the work applies entropy. In terms of contrastive learning, ✗ means t24hat the work does not apply this technique, and ✓ means that the work uses this technique.

**Table 2:** Example of a full-word mask for RoBERTa_wwm.

[Original text]	BITTER APT组织是一个长期活跃的境外网络攻击组织。
[BERT]	BITTER APT组织是一个长期活跃的[MASK] [MASK]网络攻击组织。
[RoBERTa_wwm]	BITTER APT组织是一个长期活跃的[MASK][MASK][MASK][MASK][MASK][MASK][MASK] [MASK]。

**Table 3:** Entity definition.

Entity Type	Entity Description
Admin	Professionals who manage and protect computer systems
Device	Any computing device connected to a network
Hardware	All physical components and devices of a computer
Software	Programs and data running on a computer
Operating System	The fundamental software in a computer
Service	An application or process running on a network
Network	Multiple interconnected computing devices capable of communicating and exchanging information with each other
IP	A unique numerical label assigned to each device for locating and identifying it on a network
Port	A logical channel in a network used to distinguish between different services and applications
Protector	Individuals or teams responsible for protecting computer systems
Countermeasure	Techniques employed to safeguard computer systems
Adversary	Individuals or organizations attempting to infiltrate or damage computer systems
Attack Vector	Techniques used by adversaries to gain control of computer systems
Vulnerability	Security flaws present in devices
Malware	Malicious software programs designed to cause harm to computers
Worm	Programs that destroy data by infecting computers

**Table 4:** NER performance on CDTier dataset in terms of macro-average and micro-average.

Model	Macro-Average			Micro-Average		
	Precision	Recall	F1-Score	Precision	Recall	F1-Score
EDRMRC	93.39%	90.12%	91.72%	93.48%	90.11%	91.76%
RMRC	92.58%	88.15%	90.29%	92.28%	87.93%	90.05%
RoBERTa_wwm-RDCNN-CRF	91.05%	85.96%	88.4%	90.94%	85.61%	88.19%
RoBERTa_wwm-TENER-CRF	89.78%	80.76%	84.87%	90.15%	80.89%	85.27%
BERT-Mamba-RDCNN-CRF	88.65%	76.82%	82.21%	89.03%	79.79%	84.16%
BERT-BiLSTM-GRU-CRF	71.1%	75.37%	73.09%	68.5%	74.24%	71.25%

**Table 5:** Macro-average and micro-average NER performance on MyCDTier dataset.

Model	Macro-Average			Micro-Average		
	Precision	Recall	F1-Score	Precision	Recall	F1-Score
EDRMRC	84.14%	75.01%	77.49%	78.79%	73.04%	75.81%
RMRC	83.16%	64.57%	71.92%	79.06%	64.66%	71.21%
RoBERTa_wwm-RDCNN-CRF	70.84%	59.96%	63.66%	76.92%	62.68%	68.97%

**Table 6:** Macro-average and micro-average performance on CDTier dataset.

Model	Macro-Average			Micro-Average		
	Precision	Recall	F1-Score	Precision	Recall	F1-Score
EDRRC	89.38%	93.68%	90.84%	93.12%	92.26%	92.40%
RRC	77.04%	72.54%	72.90%	89.28%	89.23%	88.98%
BERT-based method	51.00%	56.00%	48.00%	62.00%	60.00%	59.00%

**Table 7:** Performance comparison on CDTier dataset in terms of precision, recall, and F1-score.

Relation Type	EDRRC			RRC			BERT-Based Method		
	Precision	Recall	F1-Score	Precision	Recall	F1-Score	Precision	Recall	F1-Score
alias_of	92.31%	97.30%	94.74%	89.74%	90.91%	90.32%	61.00%	60.00%	60.00%
cooperate_with	100.00%	100.00%	100.00%	100.00%	33.33%	50.00%	57.00%	67.00%	62.00%
related_to	92.31%	100.00%	96.00%	84.00%	77.78%	80.77%	57.00%	74.00%	65.00%
uses	81.82%	75.00%	78.26%	76.56%	84.48%	80.33%	66.00%	57.00%	61.00%
target_at	97.37%	88.10%	92.50%	93.50%	95.90%	94.68%	62.00%	76.00%	68.00%
originated_from	100.00%	88.89%	94.12%	87.10%	79.41%	83.08%	50.00%	35.00%	41.00%
launch	60.00%	100.00%	75.00%	66.67%	100.00%	80.00%	40.00%	50.00%	44.00%
consist_of	100.00%	100.00%	100.00%	100.00%	91.67%	95.65%	50.00%	17.00%	25.00%
operating_in	100.00%	100.00%	100.00%	96.59%	94.44%	95.51%	73.00%	42.00%	54.00%
develop	70.00%	87.50%	77.78%	53.33%	50.00%	51.61%	33.00%	38.00%	35.00%

**Table 8:** Overall accuracy for RE on CDTier and MyCDTier datasets.

Model	Accuracy	
	CDTier	MyCDTier
EDRRC	92.26%	94.23%
RRC	89.23%	92.26%
BERT	60.00%	82.95%

## Data Availability

No new data were created or analyzed in this study.
